# Bettlägerigkeit bei alten Menschen – eine Literaturübersicht

**DOI:** 10.1007/s00391-024-02350-z

**Published:** 2024-09-04

**Authors:** Bianca Berger, Fabian Graeb, Manfred Baumann, Sven Reuther

**Affiliations:** 1 Fakultät Soziale Arbeit, Bildung und Pflege, Hochschule Esslingen, Flandernstraße 101, 73732 Esslingen, Deutschland; 2 Hospizstiftung Rems-Murr-Kreis e. V., Backnang, Deutschland; 3 Caritasverband für die Region Krefeld e. V. , Krefeld, Deutschland

**Keywords:** Immobilität, Mobilitätsförderung, Ortsfixierung, Einschränkung des Lebensraums, Folgen von Bettlägerigkeit, Immobility, Improving mobility, Local confinement, Restriction of living space, Consequences of bedriddenness

## Abstract

**Hintergrund:**

Insbesondere alte Menschen sind von Mobilitätseinschränkungen betroffen und können den Prozess der allmählichen Ortsfixierung bis hin zur Bettlägerigkeit durchlaufen. Letztere geht mit weitreichenden Folgen für die Personen, die pflegerisch relevant sind, einher.

**Ziele der Arbeit:**

Pflegerische Implikationen bezüglich des Phänomens der Bettlägerigkeit im Bereich der Langzeitpflege zu bündeln und Impulse für eine pflegewissenschaftliche Forschung zu geben.

**Methode:**

Recherche in relevanten Fachdatenbanken (2003–2023) anhand der Kriterien eines Scoping-Reviews.

**Ergebnisse:**

In Deutschland gibt es einen Expertenstandard zur Mobilitätsförderung, der Bettlägerigkeit und Ortsfixierung thematisiert. Aktuelle Studien zu diesen Phänomenen sind im deutschsprachigen Raum rar. Im internationalen Kontext werden diese hingegen intensiver beforscht, wobei der Fokus oft auf den Risikofaktoren für die Entstehung von Immobilität und deren negativen Folgen liegt. Die Publikationen konzentrieren sich auf die Reduktion dieser Faktoren, während die Lebensgestaltung im Bett sowie die Teilhabe und Partizipation der Betroffenen weniger Beachtung finden.

**Diskussion:**

Die Komplexität von Bettlägerigkeit wird in der aktuellen Forschung nicht umfänglich abgebildet. Zur Entwicklung einer pflegerischen Perspektive sind Forschungsvorhaben, die Aspekte des Lebens im Bett und damit die Lebenswirklichkeit bettlägeriger Menschen sowie deren Möglichkeiten der Partizipation und Teilhabe stärker berücksichtigen, zentral.

**Zusatzmaterial online:**

Zusätzliche Informationen sind in der Online-Version dieses Artikels (10.1007/s00391-024-02350-z) enthalten.

## Abstract (en)

**Background:**

Particularly older people are affected by mobility restrictions and can go through the process of gradually becoming bedridden. This can potentially lead to extensive consequences for the individuals involved, which must be considered in their care.

**Aims:**

To bundle nursing implications related to the phenomenon of being bedridden in the field of long-term care and to provide impulses for research in nursing science.

**Methods:**

Research in relevant specialist databases (2003–2023) based on the criteria of a scoping review.

**Results:**

In Germany there is a standard for maintaining and promoting mobility that addresses bed and local confinements but German language studies on these phenomena are rare. In the international context, these issues are researched more intensively, focussing on risk factors for the development of immobility and the negative consequences for those affected. The publications focus on the reduction of these factors, while less attention is paid to the organization of life in bed and the participation and involvement of those affected.

**Discussion:**

The complexity of the phenomenon is not fully illustrated in current research. In order to develop a nursing perspective research projects that also include aspects of life in bed are a central aspect in order to take greater account of the reality of bedridden people’s lives and their potential for participation and involvement.

Im Jahr 2050 werden weltweit circa 2 Mrd. Menschen 60 Jahre oder älter sein [12]. Da ältere Menschen häufiger von Mobilitätseinschränkungen betroffen sind [19], ist auch mit einer Zunahme von dauerhaft immobilen Personen zu rechnen. Der Expertenstandard *Erhaltung und Förderung der Mobilität in der Pflege* weist darauf hin, dass eine eingeschränkte Mobilität zu einer Ortsfixierung und letztendlich zu Bettlägerigkeit führen kann [11]. Gerade in Pflegeheimen wird damit das Phänomen Bettlägerigkeit von hoher und stets wachsender Bedeutung für die Pflege sein.

Im deutschsprachigen Raum sind Zegelins Arbeiten nach wie vor einflussreich [43]. Sie beschreibt Bettlägerigkeit als Prozess der „allmählichen Ortsfixierung“: von einer zunehmenden Einengung des Bewegungsradius zunächst auf einen Ort wie die Wohnung, dann auf ein Sitzmöbel bis zur „Bettlägerigkeit“. Diese kann mit negativen Auswirkungen wie Kontrakturen, Dekubitus, Reduktion der sozialen Teilhabe und der Lebensqualität einhergehen. Die Betroffenen erleben sich in ihrer selbstbestimmen Lebensgestaltung eingeschränkt und zunehmend von pflegerischer Hilfe abhängig [7]. Der Begriff der Bettlägerigkeit bleibt im internationalen Kontext dennoch unscharf und mehrdeutig (u. a. [36]).

In diesem Beitrag werden Ergebnisse einer Literaturrecherche (2003–2023) skizziert. Diese zielt darauf ab, die bekannten Aspekte von Bettlägerigkeit bei älteren Menschen innerhalb der Langzeitpflege zu erfassen und zu analysieren. Der Prozess selbst, die Verhinderung Bettlägerigwerdens, die Folgen sowie die Lebensgestaltung im Bett, inklusive pflegerischer Implikationen, werden beschrieben.

## Methode

Dem Phänomen Bettlägerigkeit wurde im Rahmen einer Literaturrecherche gemäß den Kriterien eines Scoping-Reviews [13, 32] nachgegangen. Dies beinhaltet die Entstehung und Prävention von Bettlägerigkeit, die Folgen und die Pflege bettlägeriger Personen. Das Ziel der Recherche ist es, pflegerische Implikationen anhand der gesichteten Studien zur Bettlägerigkeit aufzuzeigen. Zielgruppe waren Menschen ab 65 Jahren im Bereich der Langzeitpflege. Die Suche konzentrierte sich auf englisch- und deutschsprachige Publikationen von Januar 2003 bis April 2023 in den Datenbanken MEDLINE (PubMed), CINAHL, LIVIO und Scopus. Die Prüfung und Auswahl relevanter Artikel erfolgten durch zwei Personen anhand definierter Ein- und Ausschlusskriterien; differierende Einschätzungen wurden eingehend diskutiert (PRISMA-ScR-Flussdiagramm, Zusatzmaterial online: Anlage 1). Insgesamt wurden 41 Publikationen in die Auswertung einbezogen und analysiert. Die Kategorien wurden anhand der Fragestellung entwickelt, induktiv präzisiert und die Publikationen entsprechend zugeordnet. Unklarheiten bezüglich der Zuordnung wurden diskursiv geklärt.

## Ergebnisse

Die Darstellung und Auswertung der 41 Publikationen hinsichtlich Studiendesign, Veröffentlichung sowie der Zuordnung zu den Kategorien ist dem Zusatzmaterial online: Anlage 2 zu entnehmen. Die Darstellung der Ergebnisse erfolgt entlang der vier Kategorien: „Prozess des Bettlägerigwerdens“, „Prävention von Bettlägerigkeit“, „Folgen der Bettlägerigkeit und deren Behandlung bzw. Prävention“ sowie „Pflege von bettlägerigen Menschen“.

### „Prozess des Bettlägerigwerdens“

Dass das „Bettlägerigwerden“ prozesshaft geschieht, beschreibt Zegelin in der Studie „Festgenagelt“. Zentrales Ergebnis ist das Konzept der „allmählichen Ortsfixierung“. Sie benennt verschiedene Einflussfaktoren, die individuelle Aspekte der Betroffenen, ihre Interaktion sowie strukturelle Einflüsse umfassen, wie den Zeitdruck der Pflegenden und das damit verbundene Gefühl der Betroffenen, ihnen zur Last zu fallen. Zahlreiche Faktoren werden als veränderbar dargestellt, die also das Potenzial bieten, Bettlägerigkeit zu verhindern [43].

Studien der Forschungsgruppe um Schirghuber beschäftigen sich mit Konzeptanalysen zur Konkretion von Begrifflichkeiten, die auf die unterschiedlichen Phasen im Prozess des Bettlägerigwerdens abheben. So werden „Stuhl-“, „Bett-“ und „Hausgebundenheit“ differenziert (u. a. [36]). Allerdings wurden die neu entwickelten Konzepte noch nicht angewendet, etwa im Sinne von Prävalenzstudien.

Von Bedeutung sind solche Überlegungen vor dem Hintergrund, dass lt. einer deutschsprachigen Prävalenzstudie in Wiener Altenheimen 49,8 % der rund 3000 Teilnehmenden in der stationären Langzeitpflege bettlägerig waren [37]. Die Anzahl bettlägeriger Personen wird zumeist von den Einrichtungen unterschätzt [5]. Die Personalausstattung erweist sich als stärkster Prädiktor für die im Bett verbrachte Zeit. Bewohner in Pflegeheimen mit geringerem Personalbestand verbringen fast 6‑mal häufiger mehr als 50 % ihrer Zeit im Bett [4]. Bei zu Hause lebenden Personen zeigt sich, dass längere Perioden von Bettruhe zu einem erhöhten Hilfebedarf bei Alltagsaktivitäten führen [22]. Diese sollten vermieden werden, um eine funktionale Unabhängigkeit zu gewährleisten [23].

Für den Verbleib im Bett gibt es aus Betroffenensicht Gründe wie Krankheit, eingeschränkte Mobilität oder Müdigkeit. Sie nutzen die Zeit, um Kräfte zu schonen und sich auf künftige Aktivitäten vorzubereiten. Ein Teil der Personen nimmt den Aufenthalt im Bett wahr als Form einer partiellen Autonomie, die Kontrolle über den Lebensraum ermöglicht. Dies kann gleichzeitig zu einer Verschlechterung der Gesundheit und zu einer erhöhten Abhängigkeit führen [16]. Etwa 3 bis 5 Monate vor dem Tod nimmt die Anzahl der Tage im Bett zu. Das Zubettgehen kann somit auch als ein Hinweis für den bevorstehenden Tod betrachtet werden [24, 25].

#### Pflegerische Implikationen.

Das Wissen um den Prozess und die Einflussfaktoren ist zentral, um erste Anzeichen einer Ortsfixierung zu erkennen und pflegerische Maßnahmen zur Erhaltung der Mobilität zu initiieren. Eine einheitliche Terminologie von Begrifflichkeiten rund um das Thema Bettlägerigkeit wäre sinnvoll. Es zeigt sich auch, dass Betroffene mitunter Vorteile darin sehen, im Bett zu verbleiben, um Autonomie im Lebensraum Bett zu erleben.

### „Prävention von Bettlägerigkeit“

Ältere Menschen verlieren während längerer Inaktivitätsphasen wie bei der Genesung von Krankheiten oder Verletzungen schneller Muskelmasse als jüngere [14]. Bei längerer Inaktivität wird die Atrophie hauptsächlich durch einen Rückgang der Muskelproteinsynthese verursacht. Aufeinanderfolgende kurze Inaktivitätsphasen (< 10 Tage) spielen zudem eine wichtige Rolle bei der Entwicklung der altersbedingten Sarkopenie [41].

Körperliche Aktivität zur Förderung der Mobilität kann zwar dazu beitragen, Muskelverlusten vorzubeugen. Allerdings reichen Empfehlungen wie 2000 Schritte/Tag oder 150 min moderate Bewegung/Woche nicht aus, um Muskelverlust vollständig zu verhindern [2]. Neben altersphysiologischen Abbauprozessen spielt hier auch die Ernährung eine wichtige Rolle: um den Abbau zu bremsen, werden Nahrungsergänzungen mit Proteinen empfohlen, um so die Muskelproteinsynthese aufrechtzuerhalten [2, 14].

Pflegeeinrichtungen mit einer rehabilitativen Arbeitsweise zeigen eine geringere Prävalenz von Bettlägerigkeit und eine höhere Teilnahme an sozialen Aktivitäten [40]. Es zeigt sich, dass Immobilität nicht nur durch Krankheit oder Alter, sondern auch durch individuelle und umweltbedingte Faktoren beeinflusst wird – z. B. durch die Art und Weise, wie Pflegende ihre Rolle bezüglich Mobilität wahrnehmen und Maßnahmen individuell abstimmen [26, 34]. Dies gilt auch für die Auswahl und Bewertung von Hilfsmitteln, da deren Einsatz nicht für alle Betroffenen gleich wirksam ist [39]. Ein Bewusstsein für und das Wissen um Gefahren der Immobilität bei Pflegenden ist somit zentral. Selbstlernmodule, u. a. zur Immobilität bei älteren Menschen, werden daher als wichtiger Baustein beschrieben, um Strategien zur Verhinderung von Bettlägerigkeit einzuleiten [15].

#### Pflegerische Implikationen.

Die Publikationen weisen auf die Notwendigkeit eines multimodalen Ansatzes zur Vermeidung von Muskelverlust hin. Dieser umfasst neben Ernährung und Bewegung auch Rehabilitationspflegepraktiken. Die Pflegenden sollten eine Haltung entwickeln, die Mobilitätsförderung als Teil des pflegerischen Handelns begreift. Zudem sind spezielle Fort- und Weiterbildungsangebote zur Erhaltung der Mobilität wichtig, um Pflegenden Kompetenzen zu vermitteln, Einstellungen und Bewältigungsmuster der Betroffenen besser wahrnehmen zu können und entsprechende Maßnahmen abzustimmen.

### „Folgen der Bettlägerigkeit und deren Behandlung bzw. Prävention“

Bettlägerigkeit kann eine Vielzahl an physischen und psychischen Folgen nach sich ziehen und eine Kaskade von Abhängigkeiten und Risikofaktoren begünstigen [42]. Ein Beispiel hierfür ist der Zusammenhang zwischen Bettruhe und Schlaflosigkeit. Ältere Menschen, die 5 bis 7 Tage im Bett verbringen, erleben eine stärkere Schlaflosigkeit als diejenigen, die sich weniger oder tagsüber nicht im Bett aufhalten [17]. Darüber hinaus kann längere Bettruhe die orthostatische Intoleranz bei diesen Personen erhöhen [18] und zu einem Verlust der Haltungsmuskulatur führen [28].

Es werden weitere Auswirkungen auf das Herz-Kreislauf-System beschrieben. Ein Unterschied in der Inzidenz von venösen thromboembolischen Ereignissen zwischen immobilen und (teil-)mobilen Personen konnte jedoch nicht festgestellt werden [21]. Hautschäden wie Dekubitus sind ebenfalls mit Bettlägerigkeit assoziiert [26]. Weitere Risikofaktoren für Hautschäden können ein niedriger Knöchel-Arm-Index, das männliche Geschlecht [31] und ein schlechter Ernährungszustand sein [35]. Darüber hinaus treten bei bettlägerigen Personen häufiger Infektionen und Besiedelungen mit *S. aureus* auf [38].

#### Pflegerische Implikationen.

Es wird deutlich, dass die physischen Auswirkungen von Bettlägerigkeit gut dokumentiert sind. Pflegende sollten um die Folgen wissen und entsprechende Pflegemaßnahmen wie Prophylaxen umsetzen, um schwerwiegende Folgen zu vermeiden. Dazu gehört, individuelle Risikofaktoren einzuschätzen und diese im Blick zu behalten, um selbst oder gemeinsam mit anderen Berufsgruppen Maßnahmen zu initiieren.

### „Pflege von bettlägerigen Menschen“

Die Pflege von bettlägerigen Personen wird häufig von Familienmitgliedern übernommen, was zu erheblichen Belastungen und zu einer möglichen Überforderung führen kann. Besonders in Entwicklungs- und Schwellenländern liegt die Verantwortung für die Pflege bei den Familien. Eine fachlich unzureichende Pflege kann zu (medizinischen) Komplikationen führen [3]. Die Belastung wird durch weitere Faktoren wie den Gesundheitszustand der Pflegenden und den Grad der Abhängigkeit der bettlägerigen Person beeinflusst [6]. Wenn Pflegende sich um ihre bettlägerigen Angehörigen mit chronisch-degenerativen Krankheiten kümmern, erleben sie oft Ängste und Erschöpfung. Die tägliche Pflegearbeit, die damit verbundene Verantwortung und die Trauer über den Verlust eines „gesunden Familienmitglieds“ sind wesentliche Belastungsfaktoren [10, 30]. Angehörige sind offen für Entlastungsangebote durch Ehrenamtliche, wenn diese einer Organisation, die die Verantwortung für die Zuteilung übernimmt und die Qualität der Leistung prüft, angehören. Ein erhöhtes Verantwortungsgefühl, den Pflegebedürftigen nicht allein zu lassen, ist für die Annahme von Unterstützung hinderlich [1].

Ambient-Assisted-Living-Lösungen werden als Entlastungsmöglichkeit beschrieben; u. a. werden ein mechatronisches System zur Unterstützung eines Positionswechsels [9] oder Luftzellmatratzen mit automatischer „Wendemöglichkeit“ vorgestellt [20]. Unter anderem erweitern digitale Technologien bei bettlägerigen Personen den Zugang zu Gesundheitsdienstleistungen und machen die Bedürfnisse marginalisierter Personen sichtbar, indem Informationsangebote ortsunabhängig bereitgestellt, soziale Interaktion, emotionale Unterstützung und Kommunikation mit Pflegenden ermöglicht werden [33].

Therapien bei Bettlägerigkeit (z. B. Massagetherapien) zeigen keine signifikanten Effekte auf die Verbesserung der subjektiven Lebenszufriedenheit [27]. Pflegerische Prophylaxen hingegen sind wichtig, um gravierende gesundheitliche Folgen zu vermeiden [26]. Wenn eine Person bereits mehr als ein halbes Jahr bettlägerig ist, kann eine Sondenernährung die Lebensdauer nur um ein weiteres halbes Jahr verlängern. Dies zu berücksichtigen, könnte bei der Entscheidung für oder gegen eine Sondenernährung helfen [29].

#### Pflegerische Implikationen.

Die „Pflege von bettlägerigen Personen“ stellt für informelle und professionelle Pflegende eine erhebliche Herausforderung dar. Es ist wichtig, Angehörigen und Pflegenden Kompetenzen zu vermitteln, die sie benötigen, um die Pflege von bettlägerigen Personen durchzuführen. Individuelle Belastungsfaktoren der Angehörigen sollten mit professionell Pflegenden thematisiert werden. Die Beratung zu geeigneten Entlastungs- und Unterstützungsleistungen ist notwendig.

## Diskussion

Es wurden 41 Artikel detektiert. Die folgenden Ergebnisse, Forschungslücken und -bedarfe sind zu benennen: Obwohl das Phänomen Bettlägerigkeit mit erheblichen gesundheitlichen Risiken verbunden ist, existieren nur wenige Prävalenzstudien oder Studien, die pflegerisches Handeln explizit thematisieren. Dass es sich um ein häufig auftretendes, aber unterschätztes Phänomen handelt, ist Ergebnis der Literaturrecherche.

Der Prozess des Bettlägerigwerdens und dessen Prävention sollten aufgrund des komplexen Zusammenspiels von Pflegebedürftigen und Pflegenden stärker Beachtung finden. Dieser Prozess ist nicht nur durch physische Faktoren und das Alter bedingt, sondern Ergebnis eines hochkomplexen Zusammenspiels verschiedener Faktoren, das vom Umfeld der Person, den Pflegenden und den individuellen Bewältigungsstrategien bestimmt wird [34].

Daher sind weitere empirische Arbeiten notwendig, die über eine bloße Erforschung von Einzelmaßnahmen hinausgehen, um die komplexe Intervention „Mobilitätsförderung“ in den Blick zu nehmen und konzeptionell auf den Alltag der pflegebedürftigen Personen abzustimmen [8]. Darüber hinaus ist es wichtig, dass Pflegende Kompetenzen entwickeln, um Anzeichen und Ursachen für eine Verschlechterung der Mobilität frühzeitig zu erkennen und dieser entgegenzuwirken. Zudem sollten Studien künftig stärker auf Aushandlungsprozesse fokussieren, z. B. wenn Personen im Bett verbleiben möchten. Es ist der Frage nachzugehen, wie Partizipation und selbstbestimmte Entscheidungsprozesse der Betroffenen gefördert werden können, um Wohlbefinden und partielle Autonomie gegen die negativen Folgen des Verbleibs im Bett abzuwägen.

Tritt Bettlägerigkeit ein, müssen damit verbundene Risiken berücksichtigt und entsprechende Prophylaxen und Maßnahmen in die Pflege integriert werden. Psychosoziale Auswirkungen wie Isolation, Depression oder Angst werden nur unzureichend thematisiert, was einen Mangel an darauf abgestimmten Konzepten und Interventionen impliziert. Neben den dominant diskutierten physischen Folgen von Bettlägerigkeit sollten daher auch Fragen der Teilhabe und Partizipation zur individuellen Gestaltung des „Lebensraumes Bett“ bedacht werden. Die Entwicklung spezifischer Konzepte, die auf bettlägerige Menschen abzielen und ihre Lebenswirklichkeit berücksichtigen, u. a. die Gestaltung des Zimmers sowie die Interaktion mit bettlägerigen Personen sollten daher Gegenstand künftiger Forschung sein. Es sollte auch berücksichtigt werden, wie die Bedürfnisse dieser Personen sichtbar gemacht werden können. Hierfür sind Schulungsangebote notwendig, um Pflege- und Betreuungskompetenzen für stark mobilitätseingeschränkte und Bettlägerige zu befördern.

Formen von Bettlägerigkeit, die die Intensität des Liegens beschreiben, sind heterogen. Konsentierte Definitionen für den Bereich der Pflege sind notwendig, um einen einheitlichen Wissensstand und ein einheitliches Verständnis sicherzustellen.

## Limitationen

Spezielle Erkenntnisse der Pflegepraxis bleiben durch eine fehlende Sichtung der grauen Literatur unberücksichtigt. Deutsche und englische Publikationen aus vier Datenbanken wurden einbezogen, sodass Publikationen in weiteren Sprachen und aus anderen Datenbanken keine Beachtung finden.

## Fazit für die Praxis

- Eine Intensivierung edukativer Angebote ist notwendig, um einer zunehmenden Immobilisierung und Bettlägerigkeit entgegenzuwirken.
- Die Entwicklung von Konzepten für Bettlägerige unter Berücksichtigung von „sozialer Teilhabe“ und „Partizipation“ ist notwendig.
- Den Wunsch von Betroffenen, im Bett zu bleiben, gilt es, als partielle Autonomie anzuerkennen und zu berücksichtigen.
- Die Thematisierung von Belastungssituationen und Entlastungsangeboten für pflegende Angehörige ist wichtig.

## Supplementary Information

Anlage 1 PRISMA-ScR – Flowchart

Anlage 2: Studienübersicht und -analyse
