# Supplementary material for: Bed confinement in old people—A literature review
Source: Z Gerontol Geriatr. 2024 Sep 4;58(3):209–13. [Article in German] doi: 10.1007/s00391-024-02350-z (PMC12048456; doi:10.1007/s00391-024-02350-z)
Supplement: Supplementary file 1 — Anlage 1 PRISMA-ScR – Flowchart [file 391_2024_2350_MOESM1_ESM.docx]

#### PRISMA-ScR – Flowchart

Datenbankrecherche - identifizierte Studien

**(n = 1066)**

PubMed (n = 426)

LIVIO (n = 384)

Scopus (n = 143)

CINAHL (n = 106)

Handsuche (n=7)

**Identifikation**

Ausschluss von Duplikaten **(n = 416)**

Titel / Abstract Screening **(n = 650)**

Ausgeschlossen **(n = 400)**

Volltexte ausgeschlossen Begründung (n = 209)

Nicht verfügbar (n = 3)

Falsche Indikation (n = 44)

Zurückgezogene Studie (n =3)

Falsche Intervention (n = 24)

Falsches Studiendesign (n = 10)

Falsche Zielgruppe (n = 26)

Falsches Setting (n =61)

Zusammenfassung einer bereits einbezogenen

Studie (n = 1)

Nicht Peer-Reviewed (n=4)

Studien vor 2003 (n=33)

Volltext auf Eignung geprüft **(n = 250)**

**Screening und Eignung**

**Einschluss**

Eingeschlossene Volltexte

**(n = 41)**

Folgende Stichwörter wurden berücksichtigt:„bedridden“„bedridden patients“ „bedridden*“, „bed-rest“, „bed-bound*“ „bed-fast“, bed*. Die unterschiedlichen Schreibweisen und die entsprechenden MeSH Terms gleichermaßen. Zudem wurden Nurs* oder Care*berücksichtigt. Fokussiert wird auf die Zielgruppe Menschen 65+. Sprache: Deutsch oder Englisch. (Stand Recherche / eingeschlossene Datenbanken 04/2023). Es wurde auf alle Settings fokussiert und in einem zweiten Schritt nur relevante Publikationen für das Setting der Langzeitpflege berücksichtigt.

Ausschluss folgender Quellen: Conference abstracts, Brief overviews, Posters, Editorials and Commentaries or Letters to the Editor, Position Papers Konferenz-Abstracts, Studienprotokolle oder -ankündigungen. Ausgeschlossen wurden Studien mit den folgenden Interventionen: Medikamente, Laborstudien (Biopsien), Sedentary Behaviour, Chair- oder Homebound wenn es nicht um die Begriffsklärung der Phase im Prozess des Bettlägerigwerdens geht.

Stand 2024--07
