# Supplementary material for: Bed confinement in old people—A literature review
Source: Z Gerontol Geriatr. 2024 Sep 4;58(3):209–13. [Article in German] doi: 10.1007/s00391-024-02350-z (PMC12048456; doi:10.1007/s00391-024-02350-z)
Supplement: Supplementary file 2 — Anlage 2: Studienübersicht und -analyse [file 391_2024_2350_MOESM2_ESM.docx]

Abbildung Veröffentlichung nach Ländern

Abbildung Veröffentlichung nach Jahren

Abbildung Verwendete Studiendesigns

| **Kategorie „****Prozess des Bettlägerigwerdens“** | | | | | | |
| --- | --- | --- | --- | --- | --- | --- |
| **Autor** | | **Zielsetzung/ Ziel** | **Studiendesign** | **n** | **Setting** | **Land** |
| Bates-Jensen, Alessi, et al., 2004 | | Vergleich von Pflegeheimen mit hohen und niedrigen Raten von bettlägerigen Bewohner hins. der im Bett verbrachten Zeit des Aktivitätsniveaus. | Kohortenstudie | 451 | Pflegeheim | USA |
| Bates-Jensen, Schnelle, et al., 2004 | | Untersuchung der Auswirkungen der Personalausstattung auf die Zeit, die während des Tages im Bett verbracht wird bei Bewohnern von Pflegeheimen. | Querschnittsstudie | 882 | Pflegeheim | USA |
| Fox et al., 2009 | | Untersuchung der Wahrnehmung von Tagen, die im Bett verbracht werden, aus Sicht der Betroffenen. | Prospekt. Kohortenstudie | 46 | Einrichtungen zur Betreuung von Menschen mit chron. Krankheiten | Kanada |
| Gill et al., 2004 | | Untersuchung des Zusammenhangs zwischen Episoden von Bettruhe und Funktionseinbußen über einen Zeitraum von 18 Monaten. | Prospekt. Kohortenstudie | 680 | Gemeindenahe & häusliche Pflege | USA |
| Gill et al., 2015 | | Bewertung der Auswirkungen von Bettruhe vs. reduzierter Aktivität aufgrund von Krankheit oder Verletzung. | Prospekt. Kohortenstudie | 754 | Gemeindenahe & häusliche Pflege | USA |
| Gill et al., 2018 | | Vergleich der Aktivitätseinschränkungen am Lebensende und der damit verbundenen Symptome. | Prospekt. Kohortenstudie | 737 | Gemeindenahe & häusliche Pflege | USA |
| Gill et al., 2019 | | Bewertung des zeitlichen Verlaufs der "Bettruhe" am Lebensende und Untersuchung der Unterschiede in Abhängigkeit von Alter, Geschlecht und Todesursache. | Prospekt. Querschnittsstudie | 651 | Gemeindenahe & häusliche Pflege | USA |
| Schrank et al., 2013 | | Bestimmung der Prävalenz von Bettlägerigkeit und Ortsfixierung. | Querschnittsstudie | 3054 | Pflegeheim | Österreich |
| Schirghuber & Schrems, 2018 | | Darstellung der aktuellen Diskussion des deutschen und englischen Sprachgebrauchs für die Begriffe "ortsfixiert" und "bettlägerig". | Konzeptanalyse | - | Alle Pflegeeinrichtungen | Österreich |
| Schirghuber & Schrems, 2021b | | Analyse des Konzepts und Entwicklung einer begrifflichen Definition von „Homebound“. | Konzeptanalyse | - | Alle Pflegeeinrichtungen | Österreich |
| Schirghuber & Schrems, 2021a | | Darstellung der Belastung bzgl. Gebundenheit und Auswirkungen auf die Pflege. | Scoping Review | - | Alle Pflegeeinrichtungen | Österreich |
| Schirghuber et al., 2022 | | Überprüfung eines konzeptionellen Modells im Hinblick auf Haus- und Rollstuhlgebundenheit sowie Merkmale, Antezedenzien und Risikofaktoren. | Delphi Studie | - | Alle Pflegeeinrichtungen | Österreich |
| Schirghuber & Schrems, 2023 | | Analyse der Konzepte und Entwicklung einer begrifflichen Definition von "an den Rollstuhl gebunden“ und "bettlägerig sein“. | Konzeptanalyse |  | Alle Pflegeeinrichtungen | Österreich |
| Zegelin, 2005 | | Sensibilisierung für den Prozess des Bettlägerigwerdens, Schaffung von Wissen über die verschiedenen Ursachen und Arten der Bettlägerigkeit und Beschreibung der Faktoren, die Bettlägerigkeit beeinflussen. | Grounded Theory | 32 | Pflegeheim & häusliche Pflege | Deutschland |
| Zegelin, 2008 | | Vermittlung von Erkenntnisse über den Prozess des Bettlägerigwerdens. | Grounded Theory | 32 | Pflegeheim & häusliche Pflege | Deutschland |
| *Publikationen von Schirghuber et. und Zegelin wurden im Rahmen des Artikels exemplarisch und nicht einzeln benannt.* | | | | | | |
| **Kategorie „****Pflege von bettlägerigen Menschen“** | | | | | |  |
| **Autor** | **Zielsetzung/ Ziel** | **Studiendesign** | **n** | **Setting** | **Land** |  |
| Abarca et al., 2018 | Untersuchung der Wahrnehmungen von Pflegenden bezüglich der Nutzung von Technologie für die ehrenamtliche Entlastungspflege bettlägeriger älterer Menschen. | Ethnographische Studie | 10 | Gemeindenahe & häusliche Pflege | Chile |  |
| Bains & Minhas, 2011 | Ermittlung der Art und Weise, wie Pflegende die häusliche Pflege von bettlägerigen erwachsenen Patienten sicherstellen. | Deskriptive Querschnittsstudie | 305 | Gemeindenahe & häusliche Pflege | Indien |  |
| Bekdemir & Ilhan, 2019 | Erhebung der Belastung von Pflegekräften, die bettlägerige Personen betreuen, und der Faktoren, die die Belastung beeinflussen. | Beobachtungsstudie | 312 | Gemeindenahe & häusliche Pflege | USA |  |
| Bruno et al., 2016 | Vorstellung eines technischen Systems zur Unterstützung des Pflegepersonals beim der Positionsveränderung bettlägeriger älterer Menschen in häuslicher Umgebung durch ein mechatronisches System. | Designforschung | k. A. | Häusliche Pflege | Portugal |  |
| Campos et al., 2021 | Verstehen der Sorgen und Nöte von pflegenden Angehörigen, die bettlägerige ältere Patienten mit chronisch degenerativen Krankheiten betreuen. | Beobachtungsstudie | 10 | Gemeindenahe & häusliche Pflege | Brasilien |  |
| Futamura et al., 2008 | Evaluation des Liegekomforts bei bettlägerigeren älteren Menschen, die eine Luftkammermatratze mit automatischem Drehmechanismus verwenden. | Quasi-experimentelle Studie | 10 | Langzeitpflegeeinrichtungen | Japan |  |
| Hirakawa et al., 2005 | Bewertung der Wirkung der häuslichen Massage-Rehabilitationstherapie bei bettlägerigen älteren Menschen. | RCT | 40 | Gemeindenahe & häusliche Pflege | Japan |  |
| Kosaka et al., 2012 | Untersuchung der Überlebenszeiten nach Sondenernährung bei bettlägerigen älteren Patienten. | Prospekt. Kohortenstudie | 163 | Langzeitpflegeeinrichtungen | Japan |  |
| Mamom & Daovisan, 2022 | Untersuchung der Belastung informeller Familienpflegekräfte , die chronisch kranke, bettlägerige ältere Personen pflegen. | Struktureller Modellierungs-Ansatz | 30 | Gemeindenahe & häusliche Pflege | Thailand |  |
| Pinero de Planza, M.A. et al., 2021 | Darstellung der gemeldeten gesundheitlichen Ausgrenzung und Marginalisierung von gebrechlichen, bettlägerigen Personen, die zuhause leben. | Konsumentenforschung | 164 | Gemeindenahe & häusliche Pflege | Australien |  |

| **Kategorie „Prävention von Bettlägerigkeit“** | | | | | |
| --- | --- | --- | --- | --- | --- |
| **Autor** | **Zielsetzung/ Ziel** | **Studiendesign** | **n** | **Setting** | **Land** |
| Arentson-Lantz et al., 2019 | Untersuchung, ob täglich 2000 Schritte, mit nur wenigen zusätzlichen Aktivitäten , den katabolen Stress durch Inaktivität mildern und die Muskelgesundheit erhalten können. | Experimentelle Studie | 17 | Alle Gesundheits- und Pflegeeinrichtungen | USA |
| English & Paddon-Jones, 2010 | Die Behandlung von Muskel- und Funktionsverlusten bei bettlägerigen älteren Menschen und das Aufzeigen von Strategien zur Prävention und Rehabilitation. | Literatur Review | - | Alle Gesundheits- und Pflegeeinrichtungen | USA |
| Fletcher, 2005 | Sensibilisierung für die Gefahren von Bewegungseinschränkungen bei älteren Menschen und Optimierung von Maßnahmen zur Erhaltung und Förderung der Mobilität. | Fortbildungseinheit /-modul | - | Langzeitpflegeeinrichtungen | USA |
| Reuther, 2014 | Ermittlung von Faktoren, die die Mobilität von Pflegeheimbewohnern beeinflussen und Darstellung von pflegerischen Maßnahmen zur Verbesserung der Mobilität. | Fallanalysen | 50 | Pflegeheim | Deutschland |
| Taylor & Hoenig, 2004 | Überprüfung, ob trotz der Verwendung von Hilfsmitteln eine verstärkte Notwendigkeit für persönliche Assistenz besteht. | Kohorten-Studie | 8222 | Pflegeheim & häusliche Pflege | USA |
| Vähäkangas et al., 2008 | Analyse des Zusammenhangs zwischen den Praktiken der Rehabilitationspflege und den Qualitätsergebnissen sowie Darstellung des Zusammenhangs zwischen der Rehabilitationspflege und den Qualitätsergebnissen auf Einrichtungsebene. | Kohorten -Studie | 256 | Langzeitpflegeeinrichtungen | Finnland |
| Wall et al., 2013 | Untersuchung von Muskelatrophie bei älteren Menschen., die durch langfristige Inaktivität (mehr als 10 Tage) und kurzfristige Inaktivität (weniger als 10 Tage) verursacht wird. | Review | - | Alle Gesundheits- und Pflegeeinrichtungen | Niederlande |

| **Kategorie „Folgen der Bettlägerigkeit und deren Behandlung bzw. Prävention“** | | | | | |
| --- | --- | --- | --- | --- | --- |
| **Autor** | **Zielsetzung/ Ziel** | **Studiendesign** | **n** | **Setting** | **Land** |
| Fox et al., 2010a | Untersuchung der Auswirkungen variierender Zeiten im Bett auf die wahrgenommene Schlaflosigkeit und Tagesmüdigkeit bei Erwachsenen. | Kohorten-Studie | 67 | Einrichtungen zur Betreuung von Menschen mit chronischen Krankheiten” | Kanada |
| Fox et al., 2010b | Untersuchung der Auswirkungen von längerer Bettruhe auf die orthostatische Intoleranz bei älteren pflegebedürftigen Personen. | Prospekt. Kohorten-Studie | 65 | Langzeitpflegeeinrichtungen | Kanada |
| Gatt et al., 2004 | Untersuchung des Zusammenhangs zwischen einer dreimonatigen Immobilisierung und dem erhöhten Risiko für klinisch nachweisbare venöse thromboembolische Ereignisse. | Retrosperspekt. Kohorten-Studie | 471 | Pflegeheim | Israel |
| Hampton, 2011 | Empfehlungen für die Praxis zur Erkennung, Behandlung und Vorbeugung von Hautschädigungen bei bettlägerigen Personen (u. a. Dekubitus, Dermatitis). | Fortbildungseinheit /-modul | - | Pflegeheim | UK |
| Ikezoe et al., 2012 | Untersuchung der Auswirkungen von Alter und Inaktivität aufgrund von chronischer Bettlägerigkeit auf die Atrophie der Rumpfmuskulatur. | Fall-Kontroll-Studie | 41^[[1]](#footnote-1)^ | Pflegheim & Langzeitpflegeeinrichtungen | Japan |
| Okuwa et al., 2006 | Untersuchung der Inzidenz und Identifizierung von Risikofaktoren für Druckgeschwüre an den unteren Extremitäten bei bettlägerigen älteren Erwachsenen. | Prospekt. Kohorten-Studie | 259 | Langzeitpflegeeinrichtungen | Japan |
| Santosa et al., 2020 | Analyse der Auswirkungen von Ernährungszustand und Bettruhe auf Druck, Feuchtigkeit und Reibung sowie deren Auswirkungen auf das Auftreten von Dekubitus. | Fall-Kontroll-Studie | 60 | Pflegeheim | Indonesien |
| Silva et al., 2022 | Bestimmung der Prävalenz und der assoziierten Faktoren für die nasale, orale und rektale Übertragung von S. aureus und MRSA bei bettlägerigen Patienten und Bewohnern von Langzeitpflegeeinrichtungen. | Querschnittsstudie | 226 | Langzeitpflegeeinrichtungen | Brasilien |
| Wick, 2010 | Beschreibung der heterogenen Auswirkungen und Folgen von Bettlägerigkeit. | Fortbildungseinheit /-modul | - | Pflegeheim | USA |

References

Abarca, E., Campos, S., Herskovic, V., & Fuentes, C. (2018). Perceptions on technology for volunteer respite care for bedridden elders in Chile. *Int J Qual Stud Health Well-Being*, *13*(1), 1422663. https://doi.org/10.1080/17482631.2017.1422663

Arentson-Lantz, E., Galvan E, Wacher A, Fry CS, & Paddon-Jones D (2019). 2,000Â Steps/Day Does Not Fully Protect Skeletal Muscle Health in Older Adults During Bed Rest. *J Aging Phys Act*, *27*(2), 191–197. https://doi.org/10.1123/japa.2018-0093

Bains, P., & Minhas, A. S. (2011). Profile of Home-based Caregivers of Bedridden Patients in North India. *Indian Journal of Community Medicine*, *36*(2), 114–119. http://www.redi-bw.de/db/ebsco.php/search.ebscohost.com/login.aspx%3fdirect%3dtrue%26db%3dcin20%26AN%3d104689123%26site%3dehost-live

Bates-Jensen, B. M., Alessi, C. A., Cadogan, M., Levy-Storms, L., Jorge, J., Yoshii, J., Al-Samarrai, N. R., & Schnelle, J. F. (2004). The Minimum Data Set bedfast quality indicator: differences among nursing homes. *Nurs Res*, *53*(4), 260–272. https://doi.org/10.1097/00006199-200407000-00009

Bates-Jensen, B. M., Schnelle, J. F., Alessi, C. A., Al-Samarrai, N. R., & Levy-Storms, L. (2004). The effects of staffing on in-bed times of nursing home residents. *J Am Geriatr Soc*, *52*(6), 931–938. https://doi.org/10.1111/j.1532-5415.2004.52260.x

Bekdemir, A., & Ilhan, N. (2019). Predictors of Caregiver Burden in Caregivers of Bedridden Patients. *Journal of Nursing Research (Lippincott Williams & Wilkins)*, *27*(3), e24-e24. https://doi.org/10.1097/jnr.0000000000000297

Bruno, S., José, M., Filomena, S., Vä­tor, C., Demetrio, M., & Karolina, B. (2016). The Conceptual Design of a Mechatronic System to Handle Bedridden Elderly Individuals. *Sensors (Basel)*, *16*(5). https://doi.org/10.3390/s16050725

Campos, J. S., Anjos, A. C. Y. D., Neto S. B. D. C., & Peres, R. S. (2021). Grieves and struggles of family caregivers providing care for bedridden elderly patients affected by chronic degenerative diseases. *Invest Educ Enferm*, *39*(2). https://doi.org/10.17533/udea.iee.v39n2e09

English, K. L., & Paddon-Jones, D. (2010). Protecting muscle mass and function in older adults during bed rest. *Curr Opin Clin Nutr Metab Care*, *13*(1), 34–39. https://doi.org/10.1097/MCO.0b013e328333aa66

Fletcher, K. (2005). Immobility: geriatric self-learning module. *Medsurg Nurs*, *14*(1), 35–37.

Fox, M. T., Sidani, S., & Brooks, D. (2009). Perceptions of bed days for individuals with chronic illness in extended care facilities. *Res Nurs Health*, *32*(3), 335–344. https://doi.org/10.1002/nur.20318

Fox, M. T., Sidani, S., & Brooks, D. (2010a). Differences in sleep complaints in adults with varying levels of bed days residing in extended care facilities for chronic disease management. *Clin Nurs Res*, *19*(2), 181–202. https://doi.org/10.1177/1054773810365957

Fox, M. T., Sidani, S., & Brooks, D. (2010b). The relationship between bed rest and sitting orthostatic intolerance in adults residing in chronic care facilities. *Journal of Nursing & Healthcare of Chronic Illnesses*, *2*(3), 187–196. https://doi.org/10.1111/j.1752-9824.2010.01058.x

Futamura, M., Sugama, J., Okuwa, M., Sanada, H., & Tabata, K. (2008). Evaluation of comfort in bedridden older adults using an air-cell mattress with an automated turning function: measurement of parasympathetic activity during night sleep. *J Gerontol Nurs*, *34*(12), 20–26. https://doi.org/10.3928/00989134-20081201-09

Gatt, M. E., Paltiel, O., & Bursztyn, M. (2004). Is prolonged immobilization a risk factor for symptomatic venous thromboembolism in elderly bedridden patients? Results of a historical-cohort study. *Thromb Haemost*, *91*(3), 538–543. https://doi.org/10.1160/TH03-07-0481

Gill, T. M., Allore, H. G., Gahbauer, E. A., & Han, L. (2015). Establishing a Hierarchy for the Two Components of Restricted Activity. *J Gerontol a Biol Sci Med Sci*, *70*(7), 892–898. https://doi.org/10.1093/gerona/glu203

Gill, T. M., Allore, H. G., Gahbauer, E. A., & Murphy, T. E. (2018). Burden of Restricted Activity and Associated Symptoms and Problems in Late Life and at the End of Life. *J Am Geriatr Soc*, *66*(12), 2282–2288. https://doi.org/10.1111/jgs.15566

Gill, T. M., Allore, H., & Guo, Z. (2004). The deleterious effects of bed rest among community-living older persons. *J Gerontol a Biol Sci Med Sci*, *59*(7), 755–761. https://doi.org/10.1093/gerona/59.7.m755

Gill, T. M., Gahbauer EA, Leo-Summers L, & Murphy TE (2019). Taking to Bed at the End of Life. *J Am Geriatr Soc*, *67*(6), 1248–1252. https://doi.org/10.1111/jgs.15822

Hampton, S. (2011). Practical skin care for people who are bed-bound. *Nursing & Residential Care*, *13*(3), 132–134. http://www.redi-bw.de/db/ebsco.php/search.ebscohost.com/login.aspx%3fdirect%3dtrue%26db%3dcin20%26AN%3d104649873%26site%3dehost-live

Hirakawa, Y., Masuda, Y., Kimata, T., Uemura, K., Kuzuya, M., & Iguchi, A. (2005). Effects of home massage rehabilitation therapy for the bed-ridden elderly: a pilot trial with a three-month follow-up. *Clin Rehabil*, *19*(1), 20–27. https://doi.org/10.1191/0269215505cr795oa

Ikezoe, T., Mori, N., Nakamura, M., & Ichihashi, N. (2012). Effects of age and inactivity due to prolonged bed rest on atrophy of trunk muscles. *Nursing & Residential Care*, *112*(1), 43–48. https://doi.org/10.1007/s00421-011-1952-x

Kosaka, Y., Nakagawa-Satoh, T., Ohrui, T., Fujii, M., Arai, H., & Sasaki, H. (2012). Survival period after tube feeding in bedridden older patients. *Geriatrics & Gerontology International*, *12*(2), 317–321. https://doi.org/10.1111/j.1447-0594.2011.00805.x

Mamom, J., & Daovisan, H. (2022). Listening to Caregivers' Voices: The Informal Family Caregiver Burden of Caring for Chronically Ill Bedridden Elderly Patients. *Int J Environ Res Public Health*, *19*(1). https://doi.org/10.3390/ijerph19010567

Okuwa, M., Sanada, H., Sugama, J., Inagaki, M., Konya, C., Kitagawa, A., & et al. (2006). A prospective cohort study of lower-extremity pressure ulcer risk among bedfast older adults. *Adv Skin Wound Care*, *19*(7), 391–397. https://doi.org/10.1097/00129334-200609000-00017

Pinero de Planza, M.A., Beleigoli, A., Mudd, A., Tieu, M., McMillian, P., & Lawless, M., et al. (2021). Not Well Enough to Attend Appointments: Telehealth Versus Health Marginalisation…Digital Health Institute Summit, November 5-25, 2020. *Studies in Health Technology & Informatics*(276), 72–79. https://doi.org/10.3233/SHTI210013

Reuther, S. (2014). Mobilitätsbeeinflussende Faktoren bei Bewohnern der stationären Altenhilfe in Deutschland. *Pflege Und Gesellschaft*, *19*(2), 124–138.

Santosa, A., Puspitasari, N., & Isnaini, N. (2020). A path analysis study of factors influencing decubitus in a geriatric nursing home: A preliminary study. *Fam. Med. Prim. Care Rev.*, *22*(1), 67–70. https://doi.org/10.5114/fmpcr.2020.92508

Schirghuber, J., Köck-Hódi, S., & Schrems, B. (2022). „Nicht mehr raus können!“ Validität und Differenzierung der Konzepte Hausgebundenheit, Rollstuhlgebundenheit und Bettlägerigkeit: eine Delphi-Studie ["If you're no longer able to get out and about …" Validity and differentiation of the concepts of being homebound, wheelchair-bound and bedridden: A Delphi study]. *Zeitschrift fur Evidenz, Fortbildung und Qualitat im Gesundheitswesen*, *173*, 1–16. https://doi.org/10.1016/j.zefq.2022.07.003

Schirghuber, J., & Schrems, B. (2018). Ortsfixierung und Bettlägerigkeit im Kontext von Gebundenheit (boundedness). *Pflege*, *31*(2), 87–99.

Schirghuber, J., & Schrems, B. (2021a). The burden of boundedness and the implication for nursing: A scoping review. *Nursing Forum*, *56*(4), 950–970. https://doi.org/10.1111/nuf.12637

Schirghuber, J., & Schrems, B. (2021b). Homebound: A concept analysis. *Nursing Forum*, *56*(3), 742–751. https://doi.org/10.1111/nuf.12586

Schirghuber, J., & Schrems, B. (2023). Being wheelchair-bound and being bedridden: Two concept analyses. *Nursing Open*, *10*(4), 2075–2087. https://doi.org/10.1002/nop2.1455

Schrank, S., Zegelin, A., & Mayer, H. (2013). Prävalenzerhebung zur Bettlägerigkeit und Ortsfixierung. *Pflegewissenschaft*, *16*(4), 230–238.

Silva, L. P., Fortaleza, C., Teixeira, N. B., Silva, L., Angelis, C. D. de, & Ribeiro de Souza da Cunha, M.D.L. (2022). Molecular Epidemiology of Staphylococcus aureus and MRSA in Bedridden Patients and Residents of Long-Term Care Facilities. *Antibiotics*, *11*(11). https://doi.org/10.3390/antibiotics11111526

Taylor, D. H., & Hoenig, H. (2004). The effect of equipment usage and residual task difficulty on use of personal assistance, days in bed, and nursing home placement. *J Am Geriatr Soc*, *52*(1), 72–79. https://doi.org/10.1111/j.1532-5415.2004.52013.x

Vähäkangas, P., Noro, A., Finne-Soveri, H., & Björkgren, M. (2008). Association between rehabilitation care practices and care quality in long-term care facilities. *J Nurs Care Qual*, *23*(2), 155–161. https://doi.org/10.1097/01.NCQ.0000313765.71772.66

Wall, B. T., Dirks, M. L., & van Loon, L. (2013). Skeletal muscle atrophy during short-term disuse: Implications for age-related sarcopenia. *Ageing Res. Rev.*, *12*(4), 898–906. https://doi.org/10.1016/j.arr.2013.07.003

Wick, J. Y. (2010). Bed rest: it may not be such a good idea. *Consult Pharm*, *25*(1), 59–62. https://doi.org/10.4140/TCP.n.2010.59

Zegelin, A. (2005). "Tied down"--the process of becoming bedridden through gradual local confinement. *Pflege*, *18*(5), 281–288. https://doi.org/10.1024/1012-5302.18.5.281

Zegelin, A. (2008). 'Tied down'- the process of becoming bedridden through gradual local confinement. *J Clin Nurs*, *17*(17), 2294–2301. https://doi.org/10.1111/j.1365-2702.2007.02261.x

1. **Note**: Only elderly women (aged 85.7 ± 5.5 and 87.8 ± 6) were considered. They were divided into a group of independent older women who were able to perform daily activities independently (n = 28), and a group of dependent older women who were chronically bedridden (n = 13) [↑](#footnote-ref-1)
